# Evaluation of feline heartworm disease based on gross necropsy, serology, pulmonary histopathology, and radiographic evidence in adult shelter cats in northeastern Alabama

**DOI:** 10.1186/s13071-024-06178-9

**Published:** 2024-03-29

**Authors:** C. Thomas Nelson, Calvin M. Johnson

**Affiliations:** 1 VCA Animal Medical Center of NE Alabama, Anniston, USA; 2 Auburn College of Veterinary Medicine, Auburn, USA

**Keywords:** *Dirofilaria immitis*, Cat, Heartworm, Heartworm test/testing

## Abstract

**Background:**

: Veterinary knowledge regarding feline heartworm has been increasing significantly over the past two decades. Necropsy surveys of shelter cats have shown feline adult heartworm infection prevalence to be 5–20% of the rate in unprotected dogs; however, other studies have shown feline heartworm antibody prevalence up to 33%, reflecting higher exposure rates and potential immature adult infections. Thus, the true prevalence of feline heartworm infection is likely underestimated due to the limitations of current diagnostic techniques, inadequate testing protocols, and the high likelihood of cats exhibiting transient clinical signs or dying without confirmation of infection. Diagnosing Feline Heartworm Disease (FHWD), also referred to as Heartworm Associated Respiratory Disease (HARD), is one of the conundrums of veterinary medicine. The purpose of this study was to evaluate and characterize the occurrence of Heartworm Associated Respiratory Disease [HARD] in shelter cats, naturally-infected with * D.immitis*.

**Methods:**

Fifty shelter cats slated for euthanasia between December 2009 and June 2010 were investigated by gross necropsy, radiography, serology, and lung histopathology using techniques that have been established in experimental models of cat heartworm infection. The relationship between pulmonary vascular disease and serological markers for heartworm was also examined using correlations and statistical modeling. Serology included standard heartworm antigen test and a commonly used heartworm antibody test. Also included were heat-treated heartworm antigen test and two additional heartworm antibody tests previously evaluated on experimentally-infected cats.

**Results:**

None of the cats were heartworm antibody (HW Ab) positive on a commonly used HW Ab test used by many reference laboratories even though 20% of the study cats were heartworm antigen (HW Ag) positive on heat-treated samples. Two additional HW Ab test were positive on 26% and 22% of the study cats. The combination of heat-treated HW Ag, HW Ab tests, and histopathology indicated 34% of the study cats had HARD.

**Conclusions:**

Utilizing both, the above tests, and thoracic radiographs, enhanced the ability to predict vascular disease, possibly caused by infection with immature and adult heartworms and supported the premise that cats develop heartworm disease at the same rate as dogs.

**Graphical Abstract:**

**Figure:**
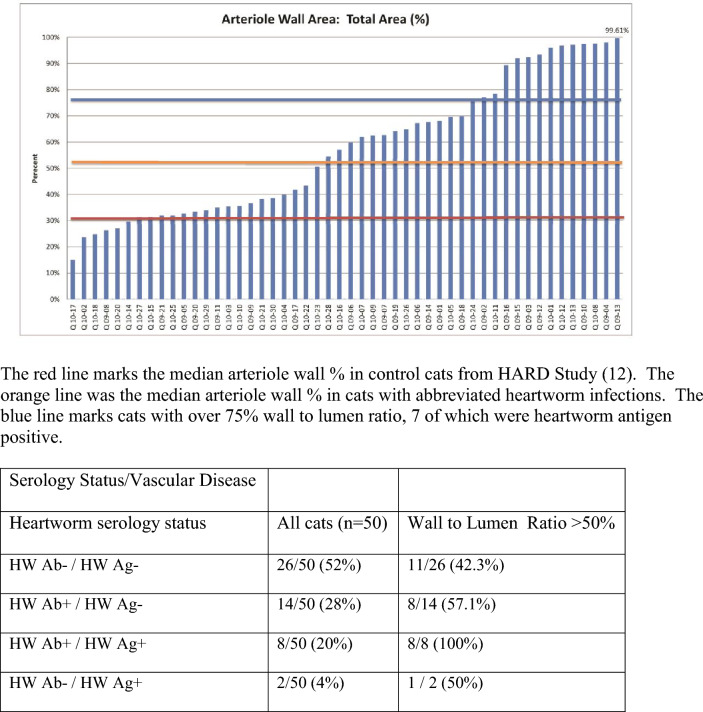

**Supplementary Information:**

The online version contains supplementary material available at 10.1186/s13071-024-06178-9.

## Background

Veterinary knowledge regarding feline heartworm has been increasing significantly over the past two decades. Although dogs are the definitive host, cats are susceptible to *Dirofilaria immitis* and are more resistant to infection with adult worms than their canine counterparts [1–3]. Heartworm has been diagnosed in all 50 states of the United States and around the world [1]. The prevalence of canine heartworm disease is geographically dependent and varies with medicalization and preventive compliance. Necropsy surveys of shelter cats have shown that feline adult heartworm infection prevalence is 5–20% of the rate in unprotected dogs in each geographical area [4]. However, other studies have shown feline heartworm antibody prevalence of up to 33% [5–8], reflecting higher exposure rates and potential immature adult infections. Thus, the true prevalence of feline heartworm infection (FHWI) is likely underestimated due to the limitations of current diagnostic techniques, inadequate testing protocols, and the high likelihood of cats exhibiting transient clinical signs or dying without confirmation of infection [1].

Female mosquitoes are the intermediate hosts, and after ingesting microfilariae while feeding on a heartworm-infected host, the larvae develop from stage 1 (L1) to the infective stage 3 (L3) over 2–4 weeks. As the mosquito feeds on the host, the L3 larvae are deposited on the skin in a drop of hemolymph and enter through the bite wound into subcutaneous tissue, molting into stage 4 larvae (L4) within a few days. Over the next 2 months, the L4 larvae migrate through muscle and adipose tissue, eventually developing into juvenile/immature worms and moving into the peripheral vein. The immature adult worms migrate via the bloodstream and through the heart, settling in the caudal pulmonary arteries as early as 70 days post-infection. In cats, most of the juvenile worms die shortly after arriving in the pulmonary arteries, and the few that mature into adult worms typically live only 2–4 years [9], compared with most juvenile worms developing into adults with a life span of 6–8 years in dogs [1, 9]. However, the feline immune response to these dead and dying juvenile worms can result in significant respiratory disease and chronic pathology, commonly referred to as heartworm-associated respiratory disease (HARD) [11–14].

FHWI is characterized by the presence of the tissue-phase larvae or vascular-phase juvenile worms or adults in cats. Feline heartworm disease (HARD) is a pathological condition of the pulmonary tissues because of a current or past infection [1, 15]. Thus, heartworm disease in cats occurs in three different stages. The first stage begins soon after the arrival of the juvenile worms in the caudal pulmonary arteries as an acute vascular and parenchymal inflammatory reaction to the presence and subsequent death of most of these worms. Due to the intense felid immune response, at this stage, most juvenile heartworm worms are killed, and few if any mature to adults. It has been postulated that the presence of pulmonary intravascular macrophages (PIMS) plays a role in this response. PIMS account for over 70% of phagocytosis in cats. In dogs, on the other hand, 80% of phagocytosis occurs in the liver by Kupffer cells [16, 17]. Nanoparticles of L3 or L4 larvae that die in tissues in the cat will be transported in the lymph and blood and taken up by PIMS. This results in the release of cytokines which may heighten the immune response. Cough or dyspnea is the resulting clinical symptom in 64% of cats. Vomiting unrelated to eating occurs in 38% of cats, and 10–20% of cats are reported to die suddenly [9, 18]. In addition, 28% of cats have no clinical signs [18]. Pulmonary parenchymal disease may be seen on thoracic radiography during this stage, and its improvement is usually seen after administration of corticosteroids, which further contributes to the frequent misdiagnosis of asthma or allergic bronchitis. If juvenile worms mature into adult worms, clinical signs may resolve due to the suppression of the host’s pulmonary macrophage activity and downregulation of the host immune response [1, 11, 12, 15].

The second stage of HARD in cats starts when the adult worm dies and the downregulation of the immune system ends [15–17]. The decomposing parasite induces a strong pulmonary inflammatory response and thromboembolism, often resulting in fatal acute lung injury, leading to a combination of respiratory distress, ataxia, collapse, seizures, hemoptysis, and sudden death in up to 20% of cats [1, 2, 15, 18]. Even a single-worm infection can induce this reaction upon the death of the worm [1, 17].

In surviving cats, hyperplastic type II alveolar cells replace the normal type I cells, leading to permanent lung injury [15, 21]. Chronic respiratory disease resulting from this lung injury is the third and final stage of HARD [15, 21]. Even after clearing the infection, cats are at risk of pulmonary arterial lesions and vascular disease. Occlusive medial hypertrophy of the small pulmonary arterioles is the main microscopic lesion observed, but changes in the bronchi, bronchioles, alveoli, and pulmonary arteries can also be present [1, 3, 12–14, 19, 21].

Less commonly, cats may present with cardiac signs such as systolic heart murmur if the worms settle in the right atrioventricular junction and interfere with tricuspid valvular function [1]. Caval syndrome is rarer in cats than in dogs, but even 1–2 worms may cause tricuspid regurgitation and heart murmur. Clinical signs such as tachycardia, vomiting, diarrhea, weight loss, and respiratory disorders may be subclinical [1, 18]. Other signs that have been noted in cats with heartworm but are uncommon include ascites, hydrothorax, chylothorax, pneumothorax, ataxia, seizures, and syncope. Despite these serious clinical implications, feline heartworm is underdiagnosed [1], and preventive regimens are utilized in fewer than 5% of cats.

Diagnosis of FHWI is often elusive due to multiple factors, including light infections consisting of only one or two worms, infections consisting of only adult male worms or immature adult female worms, infections with immature adults only, and limitations of currently available diagnostic techniques [1]. *Dirofilaria immitis* microfilariae are rarely detected in cats, because an adult male and a female worm must be present for the cat to be microfilaremic. Additionally, single-sex infections are common because cats typically have only one or two worms. When microfilariae are produced, they are only present for 1 or 2 months, at which time the cat’s immune system eliminates them and suppresses further embryogenesis [2]. For these reasons, microscopy filtration methods are not useful in detecting feline heartworm disease [1].

Although heartworm antigen (HW Ag) testing is the gold standard for identifying *D. immitis* in dogs, none of the currently available HW Ag tests are reliable to rule out heartworm infection in cats, because cats are often infected with immature worms before they die or only male worms that release very little to no detectable circulating HW Ag. Necropsy studies in shelter cats showed that 50–70% of cats infected with adult *D. immitis* had at least one female worm [22, 23]. Heartworm antigens are detectable in cats 5.5–8 months post-infection [22, 23] if female adult worms are present, and current tests identify most occult infections (i.e., adult worms are present with no circulating microfilariae) and are nearly 100% specific [1, 22]. The sensitivity of HW Ag testing in cats with mature heartworms is 50–86% [6–8, 22], which is why a negative HW Ag test does not rule out heartworm disease in cats. There may also be antigen/antibody complexes present that can interfere with results and give a false-negative reading. Heating the sample breaks down these complexes and releases the HW Ag, potentially providing a more accurate test result [24]. Heat treatment also increases the detection of HW Ag in adult male-only infections, but the sensitivity level is not near that seen in infections with female adult worms [25].

Antibody testing detects *D. immitis* infection sooner than HW Ag testing, at 2 months post-infection; however, a positive HW Ab test does not indicate current infection but only that an infection has occurred at some point [1]. One drawback is the wide range in sensitivity among the HW Ab tests, and studies in cats have shown that discordant results between HW Ab tests are common, granted the populations studied and timing of the test were not always uniform among these studies [1, 6, 26, 27]. In a necropsy study evaluating six different HW Ab tests, 21 of 31 (68%) cats with heartworms tested negative on at least one test [6]. Another necropsy study reported that out of 10 cats with heartworms, five (50%) cats had a negative HW Ab test [8]. In a study of 50 clinical cases of cats with heartworm, a 14% HW Ab false-negative rate was noted [18]. The current HW Ab tests were developed utilizing a dog model where 100 L3 larvae were used to experimentally infect cats. Unfortunately, these tests have not performed as well in real-world situations. When adult female worms are present, HW Ag tests are more reliable. It is prudent to use HW Ag and HW Ab tests together to diagnose feline heartworm, as both juvenile and adult worms can cause clinical disease in cats.

Thoracic radiography may be useful in determining the severity of heartworm disease and monitoring its progression in cats. The main radiographic finding in feline heartworm disease is an enlargement of the right caudal lobar artery, best seen in ventrodorsal view. A bronchointerstitial lung pattern that may clear spontaneously within a few months is another feature suggestive of HARD. One limitation of radiography is a lack of evidence of heartworm disease in some cases [1, 28]. In a study of 215 cats at 15 private practices in the southeastern United States, 50% of cats who had a positive HW Ag test had radiographic signs consistent with heartworm disease. Follow-up radiography in these cats showed improvement in 50% of cases and worsening of disease in 16% [29]. It is also important to note that *Toxocara cati* and *Aelurostrongylus* sp. can cause radiographic findings similar to those of heartworm and should therefore be considered in the differential diagnosis [1, 30, 31].

Echocardiography is a highly specific diagnostic tool in feline adult heartworms and can potentially detect 100% of adult heartworm infections if performed by an experienced sonographer who is able to follow the caudal pulmonary arteries to their bifurcation within the lung fields and if several worms are present [27, 32, 33]. Although heartworms are typically found in the main and right lobar branch of the pulmonary artery, it is still necessary to examine all arteries because worms may occupy only one or two sites and may escape detection [1]. Adult heartworms are also relatively long compared with the length of the pulmonary arteries in cats, so there is a good chance of finding heartworms in cats extending from peripheral branches into proximal segments where they can be visualized [1, 32]. Due to the peculiarities of feline heartworm pathophysiology and the limitations of currently available diagnostic methods, multiple tests repeated on several occasions may be required to identify this infection in cats.

Multiple studies have been conducted on cats experimentally infected with heartworms, evaluating HW Ag and HW Ab tests, radiographic findings, and pulmonary pathology [3, 11–13]. There are also multiple studies on necropsied shelter cats, determining the prevalence of adult heartworm infections, HW Ag/HW Ab status [6–8], and the extent of pulmonary pathology [19] There are even studies correlating clinical signs of heartworms in cats with serological and radiographic findings [26, 29]. Each of these types of studies has furthered our knowledge of feline heartworm disease, but each has been lacking. Experimental infections fail to mimic real-world situations. Necropsy of euthanized shelter cats lacks ante-mortem blood samples and radiographs, and surveillance of clinical patients is missing necropsy confirmation of heartworm infection and histopathology. This study was designed to bring all these facets together in naturally infected cats in an endemic area. The purpose of this study was to evaluate and characterize the occurrence of HARD in shelter cats naturally-infected with D. immitis.

### Methods

Cats were investigated by gross necropsy, radiography, serology, and lung histopathology using techniques that have been established in experimental models of cat heartworm infection. The relationship between pulmonary vascular disease and serological markers for heartworm was also examined using correlations and statistical modeling.

### Animals

A population of 50 adult cats (27 female and 23 male) estimated to be between 1 and 7 years old from a local animal control shelter that were slated for euthanasia were evaluated in this study. Between December 2009 and June 2010, selected cats from the shelter were studied at the Animal Medical Center of Northeast Alabama (Anniston, AL). This project was approved by the director of the animal control shelter and the medical director of the Animal Medical Center of Northeast Alabama, and the study design was reviewed and approved by the Institutional Animal Care and Use Committees of Pfizer Animal Health (now Zoetis).

### Data collection

Immediately upon arriving at the clinic, cats were sedated with an intramuscular dose of dexmedetomidine 0.02 mg/kg (Dexdormitor, Zoetis, Kalamazoo, MI, USA), butorphanol 0.2 mg/kg (Torbutrol, Zoetis, Kalamazoo, MI, USA), and ketamine 5 mg/kg (Ketaset, Zoetis, Kalamazoo, MI). Ventrodorsal and right lateral thoracic radiographs were obtained under sedation, and peripheral blood was drawn to obtain approximately 30 ml of serum for serology. Cats were then humanely euthanized according to the American Veterinary Medical Association Guidelines for Euthanasia using pentobarbital 86.7 mg/kg and phenytoin 11.11 mg/kg given intraperitoneally (Euthasol, Virbac, Fort Worth, TX, USA). Necropsy was performed with collection of the heart and lungs for histopathological studies. The heart, pulmonary arteries, and all lung lobes except the right caudal lung lobe were dissected, and all heartworms were collected from those animals that had them and counted. The right caudal lung lobes were perfused with 10% formalin via the main bronchus to an inflation pressure of 15 cm H_2_O and tied off to maintain expansion for processing. A transverse full-thickness sample from each lung was collected and processed for histopathology. Radiographs and histopathological structures were evaluated by a board-certified radiologist and pathologist who were blinded to the heartworm serological status and data profile of the cats.

### Data evaluation

Data reported in this paper include results from thoracic radiographs, lung histopathology, and serology. Lateral and ventrodorsal view radiographs were submitted to a boarded radiologist who was blinded to the results of necropsy, serology, and histopathology. Thoracic radiographs were graded for severity of lung lesions using a 0–3 scoring system (0 = normal and 3 = severe lesions), as described previously [29]. Radiographs were graded for pulmonary artery size, bronchial pattern, interstitial pattern, and mixed bronchointerstitial–alveolar pattern. Any abnormalities in the heart, pleura, or diaphragm were also recorded. The images were reviewed on two different occasions, and images with discordant results were evaluated a third time, and the final score was tabulated.

Histological examinations of the right caudal lung lobes were conducted on fixed-perfused right caudal lung lobes that were sectioned and stained with hematoxylin and eosin (H&E) and α-smooth muscle actin (HSRL Inc., Mount Jackson, VA, USA). Morphometric analyses were performed for arterioles and bronchioles as described previously [12]. For each of the 50 slides, a minimum of five random bronchioles and their corresponding arterioles were photographed with the ×10 objective, using Q Capture Pro software. Measurements on digital images were made for each cat using ImageJ software (National Institutes of Health [NIH], http://rsb.info.nih.gov/ij/) by tracing the outer wall and lumen of each arteriole and then calculating the area of the lumen, the area of the wall, and the total area of the wall and lumen. Data were analyzed by calculating the ratio of the arteriole wall area to total area, expressed as a percentage. Based on Dillon’s work, any arteriole wall ratio >  50% was considered to have pulmonary vascular disease [12].

Serological evaluations were performed on banked frozen serum samples using commercially available or previously published assays for HW Ag and HW Ab (IDEXX Laboratories, Inc., Westbrook, ME, USA) [34, 35]. Briefly, HW Ag testing was performed using a microtiter plate enzyme-linked immunosorbent assay (ELISA) that is commercially available through a veterinary diagnostic laboratory (Heartworm Antigen by ELISA and Heartworm Antigen with Heat Treatment, IDEXX Laboratories, Inc., Westbrook, ME, USA). Heartworm Ab testing was performed using two different heartworm-specific recombinant antigen targets (HWAg-1 and HWAg-2) in an indirect ELISA format and a 1:400 dilution of the serum sample. HW Ab was also measured using a commercially available microtiter plate assay (Synbiotics Corporation, Zoetis, Florham Park, NJ, USA).

### Statistical analysis

All statistical analyses were performed with SAS/STAT software, version 9.4 (SAS Institute Inc., Cary, NC, USA). To conservatively evaluate the predictive power of combined tests (ranked radiographs, HW Ab−, and HW Ag− test scores) on the arteriole score, a marginal linear mixed-effects model with an empirical sandwich-based robust covariance estimator was used to analyze the data [36]. Model goodness of fit was assessed based on the information criteria (Akaike information criterion [AIC], Bayesian information criterion [BIC], corrected AIC [AICc]), proportion of explained variance (R^2^), and Chi-square test for the model covariance structure [37]. Model residuals were plotted and checked for independence on the linear predictor and normality of distribution. In addition to a type III test for statistical significance, the importance of factor inclusion in the model was verified using the likelihood ratio test (LRT).

Further statistical analyses examined the impact of adding radiographic scores to the data model for predicting the degree of arteriole occlusion.

## Results

### Necropsy

Adult heartworms were found in 3/50 cats (6%) at necropsy survey. Two of these three cats had adult worms, and one had worm fragments (Table 1).

**Table 1:** Adult heartworms were found in 3/50 cats (6%) at necropsy

Cat number	Viable adult worms (male, female)	Dead worms or fragments
Q10-12	1 (1 female)	0
Q09-03	2 (1 male, 1 female)	0
Q10-09	0	2 fragments

**Table 2:** Results of serological testing (number of cats positive for the test/number of cats tested; *n* = 50)

Test	No. of cats with positive test (%)
Symbiotic antibody (ELISA)	0/50
HW antibody-1 (ELISA)	13/50 (26%)
HW antibody-2 (ELISA)	11/50 (22%)
HW antigen (PetChek)	3/50 (6%)
HW antigen with heat treatment (ELISA)	10/50 (20%)

### Serology

Standard HW Ag (Heartworm Antigen by ELISA, IDEXX Laboratories, Inc., Westbrook, ME, USA) and HW Ab (Synbiotics Corporation, Zoetis, Florham Park, NJ, USA) commonly utilized in veterinary practices were performed on frozen serum samples obtained from the 50 cats. Three cats were HW Ag-positive (HW Ag+), and no cats had HW Ab initially detected. Based on these results utilizing routine HW Ag and HW Ab tests, one would presume that 6% of this cohort had FHWI and disease. When serum samples were heat-treated with an established protocol [34], seven additional samples were positive, for a total of 10 HW Ag+ cats (20%). The research-purpose antibody assays HW Ab1 and HW Ab2 were positive in 26% and 22%, respectively. Sixty percent of the cats tested negative on all HW Ab and HW Ag tests. Thirty-two percent were positive on at least one of the HW Ab1 or HW Ab2 assays. The two cats in which a whole worm was recovered were HW Ag+, and the cat with worm fragments was HW Ag-negative (HW Ag−). As no cats were positive on the Synbiotics HW Ab test, it was not included in the analysis (Table 2).

**Fig. 1:**
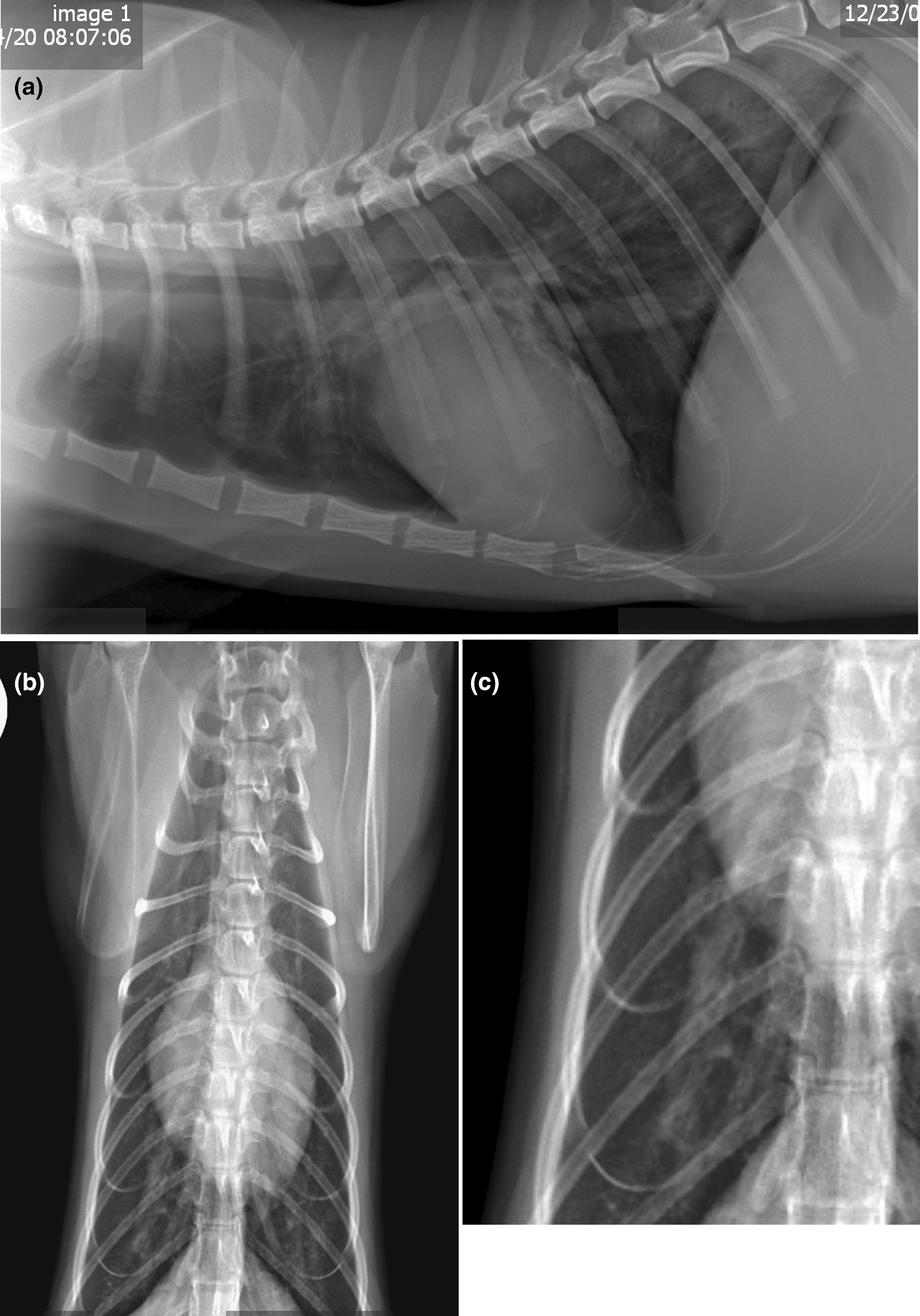
Thoracic radiographs of a cat with heartworm infection and bronchointerstitial lung pattern. **A** Right lateral view. **B** Ventrodorsal view. **C** Closer view of the enlarged right lobar pulmonary artery in the ventrodorsal view

### Radiology

Radiographs were scored on a scale of 0 to 3, as described previously (Table 3) [29]. Thirteen of the 50 cats had abnormal radiographic scores of 1 or above. Only one cat had a radiographic score of 3, and it was neither HW Ag+ nor HW Ab+. All four of the cats with a radiographic score of 2 were either HW Ag+ or HW Ab+ on at least one test, and five of the eight cats with a radiographic score of 1 were either HW Ag+ or HW Ab+ on at least one test. There were 25 cats with a radiographic score of 0.5. This score, while consistent with feline heartworm disease, is not specific. Twenty-five of the cats had a radiographic score of 0.5, and six of these were either HW Ag+ or HW Ab+ on at least one test. All 10 cats that were HW Ag+ on the standard or heat-treated HW Ag test had some degree of radiographic findings consistent with feline heartworm disease. An example of a radiographs from a heartworm positve cats are shown in Fig. 1.

**Table 3:** Radiographic scores and heartworm antigen and antibody results

Radiographic score	Radiographic score definition^a^	Criteria	Number of cats, % (*n* = 50)	Cats Ag+ or Ab+ on one or more tests, %
3	Strongly indicative of FHD	Caudal lobar artery enlargement with or without pulmonary or other abnormalities	1/50 (2%)	0/1 (0%)
2	Moderately indicative of FHD	Caudal lobar artery enlargement with or without pulmonary or other abnormalities	4/50 (8%)	4/4 (100%)
1	Mildly indicative of FHD	Caudal lobar artery enlargement with or without pulmonary or other abnormalities	8/50 (16%)	5/8 (63%)
0.5	Consistent with but not specific for FHD	Increased broncho-interstitial opacity only	25/50 (50%)	6/25 (24%)
0	No radiographic sign of FHD		12/50 (24%)	4/12 (33%)

Ab, antibody; Ag, antigen; FHD, feline heartworm disease

^a^Brawner et al. 2000

**Table 4:** Serology/vascular disease

Heartworm serology status	All cats (*n* = 50)	> 50%
HW Ab−/HW Ag−	26/50 (52%)	11/26 (42.3%)
HW Ab+/HW Ag−	14/50 (28%)	8/14 (57.1%)
HW Ab+/HW Ag+	8/50 (20%)	8/8 (100%)
HW Ab−/HW Ag+	2/50 (4%)	1/2 (50%)

### Lung histopathology

#### Arteriole wall

**Fig. 2:**
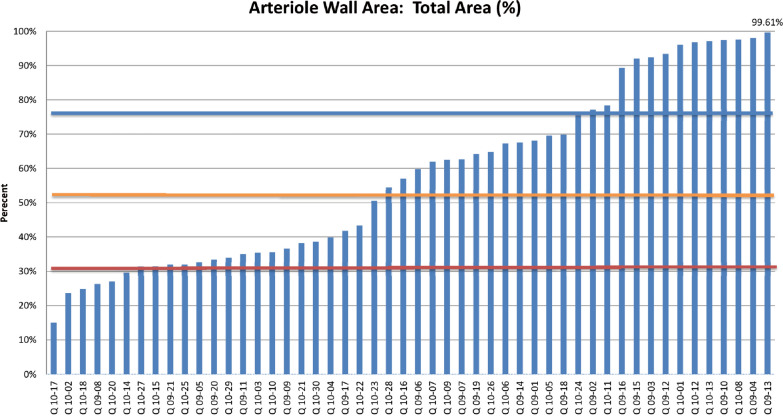
Arteriole wall area. The red line marks the median arteriole wall percentage in control cats from the HARD Study. The orange line represents the median arteriole wall percentage in cats with abbreviated heartworm infections. The blue line marks cats with over 75% wall-to-lumen ratio, seven of which were heartworm antigen-positive

**Fig. 3:**
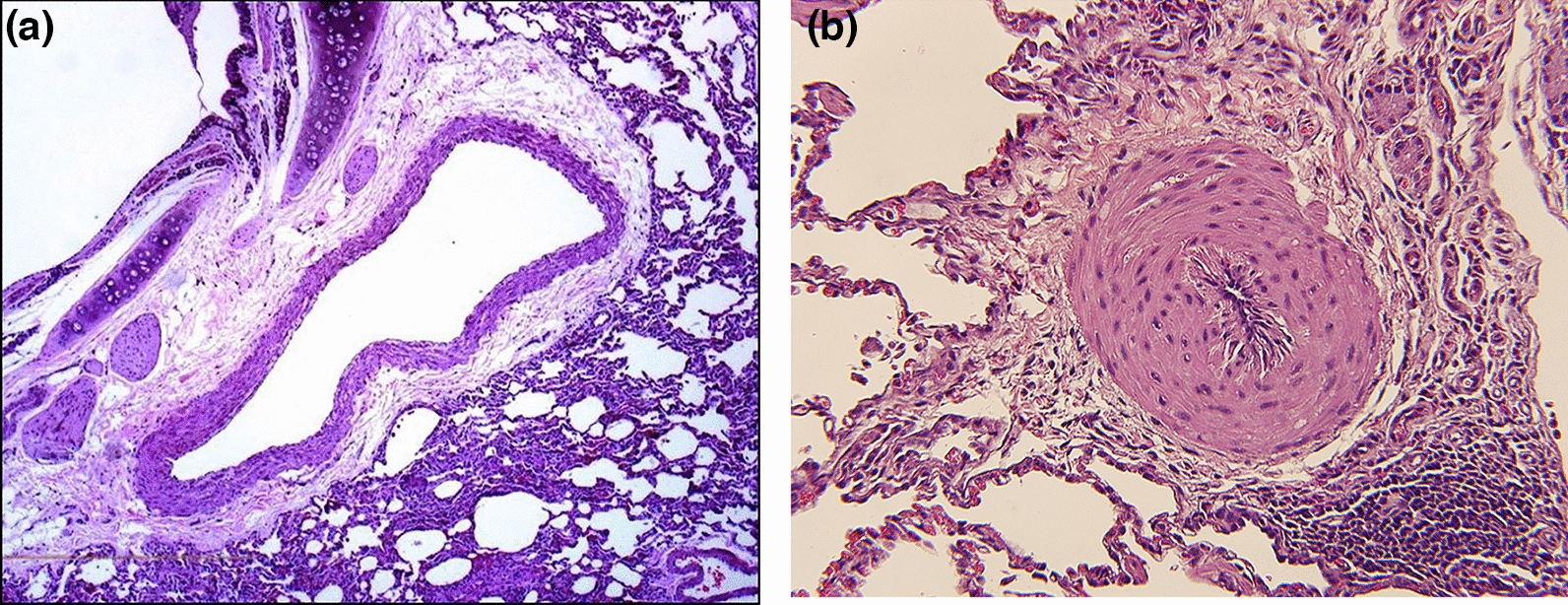
Histological sections of fixed perfused right caudal lung lobes from selected cats showing a normal vessel and one with occlusive disease: **A** a normal pulmonary arteriole with a 20% wall-to-lumen ratio, **B** a diseased pulmonary arteriole with a > 90% wall-to-lumen ratio

Pulmonary vascular disease caused by mature adult heartworms and immature heartworms has been previously described previously [13, 14, 19, 20, 38]. The arteriole wall to total area ratio ranged from 26.31% to 99.61%. Figure 2 shows the ratio of the graft of the arterial wall to total area in this cohort of cats. There are three groupings noted on this graft; the first around 30%, the second starting at 50%, and the third at 75%. Utilizing these criteria, 28 of 50 (56%) cats had vascular disease. HW Ag or HW Ab was positive in 57% (16 of 28) of cats with vascular disease and in 40% (20 of 50) of all cats studied. An example of a normal pulmonary arteriole and a diseased vessel is shown in Fig. 3.

**Fig. 4:**
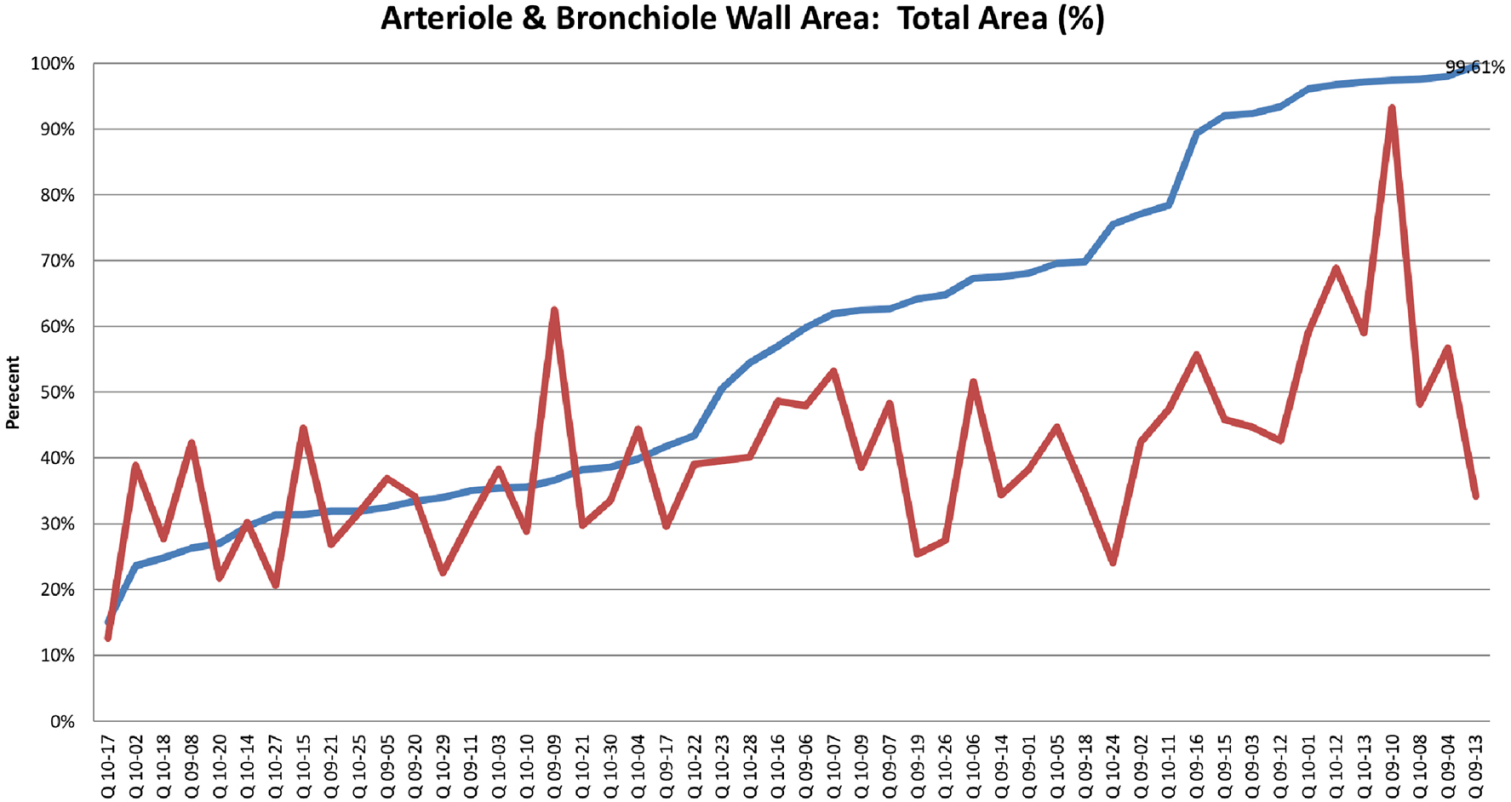
Arteriole and bronchiole wall comparison. The blue line represents the arteriole wall area-to-lumen ratio. The red line is the bronchiole wall area to lumen ratio

#### Bronchioles

The bronchiole wall area to total area ranged from 93.24% to 12.60% in this cohort of cats. There was not the same degree of correlation with serology as was noted on arteriole walls (Fig. 4).

When the serological data were compared with the histopathological findings of the pulmonary arterioles, a distinct correlation was noted. Of the eight cats that were both HW Ag+ and HW Ab+ on at least one HW Ab test, all had demonstrable arterial disease. Of the cats that were antibody-positive but HW Ag−, 57% had vascular disease. Nine out of 10 HW Ag+ cats had a ratio of arteriole wall thickness to total area > 50% (Table 4).

### Statistical modeling

Statistical analysis was challenging due to a large difference in scales and substantial deviation from normality of some of the parameters measured. It was decided to stick with a nonparametric analysis using ranks instead of actual score values. The Spearman correlation coefficient and corresponding *P*-value for the correlation of different predictors of the arteriole score were then calculated.

The HW Ab1 and HW Ag results (with heat treatment) exhibited modestly strong and statistically significant correlations with the arteriole wall thickness utilizing the Spearman correlation coefficients (Additional file 1).

A positive result on HW Ab1 correlates with arteriole occlusion to the same extent as does bronchiole occlusion, in terms of the Spearman correlation coefficient. Equally important, no correlation was detected with the other antibody assays (Table 5).

**Table 5:** Spearman correlation between arteriole occlusion and other results

Variable	Spearman correlation coefficient	*P*-value
HW Ag (untreated)	0.33	0.020
HW Ag (heat-treated)	0.38	0.007
HW Ab1	0.55	< 0.0001
HW Ab2	0.04	0.786
Bronchiole occlusion	0.59	< 0.0001
Radiographic score	0.47	0.0006

HW Ag, heartworm antigen; HW Ab, heartworm antibody

Type III tests for statistical significance suggest that three serological test results (HW Ab1, HW Ab2, and HW Ag with heat treatment) are informative predictors of arteriole disease in this shelter cat cohort. A good fit of the data can be made with three heartworm serological test results (no over-dispersion (*χ*^2^/DF = 1), and no model covariance structure mismatch (LRT, *P* = 0.58) is observed (see Additional file 2 for the model fit details). All three test results together explain 42% of arteriole variance (*R*^2^ = 0.42), and there is ~ 60% agreement between the model-predicted and actual arteriole ranks (concordance correlation = 0.59). Chi-square and *F*-test results agree, indicating statistical robustness of this conclusion (Table 6).

**Table 6:** Type III tests of fixed effects

Effect	Num DF	Den DF	Chi-square	*F*-value	Pr > ChiSq	Pr > F
HW_Ab1	1	46	32.62	32.62	< .0001	< .0001
HW_Ab2	1	46	7.34	7.34	0.0067	0.0094
HW_Ag (heat-treated)	1	46	4.02	4.02	0.0449	0.0508

Adding radiographic data to serological heartworm test results significantly improves model fit (AIC drops from 381 to 374; log likelihood LRT *χ*^2^ = 7.39, DF = 1, *p* = 0.007) and increases the explained portion of arteriole variance to 53%. Agreement between the arteriole observed and model-predicted ranks improved to almost 70% (concordance correlation, see Additional file 2).

The inclusion of the radiographs variable that appeared very significant (*P* < 0.005) by type III tests undoubtedly boosts the predictive power of the model: the proportion of model-explained arteriole variance is increased to over 50%, and agreement (concordance correlation) between the arteriole observed and model-predicted ranks is improved to almost 70%.

Interestingly, even though the inclusion of radiographs makes the HW Ag effect not statistically significant by type III tests, HW Ag remains an important predictor because it still significantly affects the model fit (by the LRT, *χ*^2^ = 4.92, *DF* = 1, *p* = 0.027). Apparently, radiographs took a considerable amount of predictive power out of HW Ag (Table 7, while HW Ab1 and Ab2 predictors were barely affected).

**Table 7:** Type III tests of fixed effects

Effect	Num DF	Den DF	Chi-square	*F*-value	Pr > ChiSq	Pr > F
HW_Ab1	1	45	31.75	31.75	< .0001	< .0001
HW_Ab2	1	45	10.76	10.76	0.0010	0.0020
HW_Ag (heat-treated)	1	45	0.09	0.09	0.7695	0.7708
Radiographs	1	45	8.95	8.95	0.0028	0.0045

## Discussion

Post-mortem examination of 50 shelter cats revealed an adult heartworm infection rate of 3/50 (6%). This finding is consistent with previous shelter studies conducted in the southern United States [6, 8, 39]. Two of these cats were HW Ag+ on a standard HW Ag test routinely performed at veterinary reference labs. All 50 cats were HW Ab− on a commercial antibody test (Synbiotics) routinely used by veterinary reference laboratories, despite the confirmed presence of adult worms in three cats. When feline HW Ab tests were first introduced, the sensitivities were reported to be ≥ 97% in detecting adult heartworm infections [40, 41]. None of the tests from the past or the tests available currently have performed as well in real-world settings. Snyder reported sensitivity ranging from 31.6% to 89.5% on multiple HW Ab tests conducted on blood samples from necropsy-confirmed samples in reference laboratories and in-house test kits [6]. Nelson reported 50% sensitivity on necropsy-confirmed samples conducted at a reference laboratory (Heska) [8]. A serological screening of 100 cats from Florida shelters found 17% HW Ab+ on the Heska Solo Step point-of-care test as compared with 2% HW Ab+ at a commercial laboratory that utilizes the Synbiotics test [27].

To understand the disparity in feline heartworm antibody test results, one needs to be aware that historically not all tests were looking for antibodies to the same HW Ag. While all heartworm HW Ag tests detect the same somatic glycoprotein from the heartworm’s reproductive tract, the feline heartworm antibody tests were designed to detect a single cuticular HW Ag, a combination of cuticular HW Ags, or a combination of cuticular and somatic HW Ags. Some common cuticular HW Ag targets used are Di5, rDi22, and rDiT33 [42–45]. These tests were originally designed to diagnose adult heartworm infections and were validated utilizing a canine heartworm experimental infection model. In this model, 40–100 L3 larvae (typically 50) are injected subcutaneously into dogs. In the cat, 100 L3 are used to ensure that enough cats are infected for the study to be valid, as the Food and Drug Administration (FDA) requires 60% of the control cats to have two or more adult worms. While this protocol will result in 80% of cats developing adult heartworm infections, it is not a reflection of what occurs in nature, as exposure rates are much lower, as Genchi reported on the attraction of and feeding habits of mosquitoes to dogs and cats [46]. He showed that a 28-kg mixed-breed male dog attracted four times as many mosquitoes as an 8-kg male cat. Furthermore, the mosquitoes attracted to the dog were three times more likely to feed, resulting in a 12-fold higher exposure rate. A trickle infection where five L3 are given weekly over a 5-week period would be better suited to validate a feline heartworm antibody test.

As antibody response is generally proportional to antigenic stimulation, the lower sensitivity noted in real-world situations should be expected. Unfortunately, this has led to under-diagnosis of HARD, which subsequently minimizes the perceived risk of heartworm disease in cats. As previously stated, the current heartworm antibody tests were designed to detect adult heartworm infections, and it has been documented that these tests fail to obtain an acceptable confidence level in naturally infected cats. We now know immature heartworms can cause the same type of pulmonary pathology as adult heartworm infections [11–14]. What we do not know is how many HARD diagnoses are being missed due to poorly performing tests.

Hypertrophy of the pulmonary arteriole walls in shelter cats that were HW Ab+ but did not have adult heartworm infections was reported [19], and later it was demonstrated in experimentally infected cats that immature heartworms caused pulmonary arterial disease [13, 14]. These studies utilized the same methodology to determine the arteriole wall ratio as was employed in this study. Dillon reported an average arteriole wall ratio of 33.2% in his control group and 52.8% in his HW Ab+ without adult heartworms group [12]. As previously stated, an arteriole wall ratio > 50% was considered to indicate pulmonary vascular disease. Utilizing these criteria, 28 of 50 (56%) cats had vascular disease.

The HW Ab1 test was positive in 13 cats, which equated to 26% of the test subjects. Twelve of these cats had arteriole wall to total area ratios greater than 50% and thus had vascular disease. HW Ab2 was positive in 11 cats, including three cats not detected by Ab1. One of these three was also HW Ag+ on a heat-treated sample and had a 68% arteriole wall ratio. Three cats were HW Ab2+ that did not have vascular disease. In all, 16 cats were HW Ab+ on one of the two additional antibody tests, and 13 of these (81.25%) had vascular disease. Twenty-eight cats had vascular disease, and 16 (57.14%) of these were positive on at least one HW Ab or HW Ag test.

As these were shelter cats and not laboratory-bred specific-pathogen-free cats, they were likely previously exposed to or infected with other parasites. *Toxocara cati* has been shown to cause similar vascular lesions in experimentally infected cats [30], and 25% of all shelter cats have been shown to be infected with *T. cati* [47, 48]*.* This could possibly explain the 12 cats (24%) with vascular lesions that were not positive on any of the HW Ab or HW Ag tests. It is also possible that the tests utilized in this study were not sufficiently sensitive or that not enough time had passed for the HW Abs to have dissipated. In Dillon’s experimental study, 50% of the cats with abbreviated infections had seroconverted to HW Ab− by 8 months [12] post-infection, and all had seroconverted to negative by 18 months [14]. The HW Ab1 and HW Ab2 tests were evaluated on experimentally infected cats (100 L3) and were shown to detect antibodies in 12/12 and 11/12 cats in the study, indicating 100% and 92% sensitivity, respectively; however, specificity was not reported. In the cohort of cats reported in this paper only one of the 13 cats that were considered positive on HW Ab1 did not have vascular disease, suggesting high specificity.

Radiographs have been shown to correlate with clinical signs and serology results. In a clinical study of 215 cats exhibiting signs of heartworm disease, 58% of cats with radiographic signs were HW Ab+ on either Heska’s feline heartworm antibody test, which uses DiT33 as its target, or the since-discontinued test offered by Animal Diagnostic, which used a combination of cuticular and somatic antigens [29]. In this study, of the 13 cats with the most significant radiographic scores, eight were either HW Ab+ on one of the two tests, HW Ag+ on a standard Ag test or heat-treated sample, or positive on multiple tests. Seven of the 10 cats with a positive heat-treated HW Ag test had radiographic scores of 1, 2, or 3. The other three Ag+ cats had radiographic scores of 0.5, which is consistent with feline heartworm disease but not specific. There were 23 cats with a radiographic score of 0.5 or higher that were negative on all serology tests. As stated previously, these were shelter cats, and a significant number also had typical upper respiratory diseases common in animal control shelter settings. These, along with the possibility of *T. cati* infections or previous heartworm infections where antibodies have dissipated, are possible explanations for the large number of cats with radiographic signs.

The initial serology results of three HW Ag+ cats for a 6% incidence were significant, but finding seven more HW Ag+ after heat-treating the samples for a 20% incidence was more than eye-opening. Multiple studies [49–52] have been conducted on both necropsy-confirmed blood samples from dogs and unconfirmed blood samples from shelter dogs, showing that heat treatment of samples improves the sensitivity of the HW Ag test, resulting in a higher positivity rate. Heat-treating of serum samples is a type of immune complex dissociation which breaks antigen–antibody complexes, allowing now unbound HW Ag to be detected. A small study of six confirmed heartworm-positive cats with low worm burden (mean = 2.0) tested cats with four different HW Ag tests, which resulted in either zero or one positive test [53]. After heat-treating serum samples, five or six cats were HW Ag+. A larger study of shelter cat samples had similar findings, with a three- to 10-fold increase in the HW Ag positivity rate following heat treatment of serum samples [54]. There was a slight decrease in specificity from 97.8% to 96.1% noted in confirmed canine blood samples that were heat-treated, suggesting that heat-treating samples is unlikely to lead to false-positive results [55]. In the seven additional cats that developed a positive HW Ag+ test after heat-treating samples, it must be noted that no adult heartworms were found in the heart or pulmonary arteries. There was one additional cat that was HW Ag+ on a standard HW Ag test that also did not have any adult heartworms identified on necropsy. These findings might be due to the fact that the right caudal lung lobes from all 50 cats were not dissected but were fixed-perfused for histopathological studies. Cats are also more likely to have ectopic infections [2], but necropsy was limited to the heart and lungs, and other locations were not thoroughly examined. Research on HW Ag–antibody complexes and their role in heartworm diagnostics is ongoing, and there is still much to learn, such as how long HW Ag–antibody complexes remain in circulation.

The base HW Ab+ rate for the southeastern region of the United States was previously reported to be 20–25% [6, 8]. The initial HW Ab test (Synbiotics) run on this cohort of shelter cats was 0% despite three confirmed adult heartworm infections. This was disconcerting, as this test is the most commonly used HW Ab test by veterinary practitioners. A high incidence of false-negative results reinforces the commonly held narrative that heartworms do not pose a significant health risk to cats. An overall lack of knowledge and understanding of the limitations of current heartworm tests available for cats has stymied the acceptance of the need for routine administration of heartworm preventives.

Statistical analysis used a conservative approach running a nonparametric model using ranks instead of actual values. While this approach reduces the predictive power of the model, it still explained over 40% of the arteriole variance. About 60% concordance correlation was found between the model-predicted and actual arteriole ranks. There were also no discrepancies found between the model-based and empirical covariance structures (variance–covariance concordance correlation = 0.96, Chi-square test for zero hypothesis that covariance structure is correct, *P* = 0.58, see Additional file 2). Inclusion of the radiographs variable that appeared very significant (*P* < 0.005) by type III tests boosted the predictive power of the model. The proportion of model-explained arteriole variance increased to over 50%, and agreement (concordance correlation) between the arteriole observed and model-predicted results improved to almost 70%.

Combining HW Ag and HW Ab tests has long been advocated for increasing the chances of diagnosing feline heartworm disease [6, 7, 39]. The findings of this study further support this narrative. If the only test run on this cohort of cats was the Synbiotics heartworm antibody test, one would be led to believe that no cats had heartworm disease. If a standard HW Ag test had been run in conjunction with the HW Ab test, 6% of the cats would then have had a diagnosis of heartworm disease. Adding a heat-treated HW Ag test increased the incidence of heartworm disease to 20%, and the additional heartworm antibody tests strongly support a heartworm disease incidence of 34% in this cohort of shelter cats from northeast Alabama. Shelter studies have reported a heartworm incidence of 28–34% in dogs in the southeastern United States [26, 56]. A necropsy survey of dogs from the same shelter as the cats reported in this study revealed that 30% of the dogs had adult heartworm infections [57]. This supports the supposition that cats are infected at the same rate as dogs in each geographical region [26, 58, 59].

## Limitations

There are several limitations to this study. The first is a lack of clinical history, as this is a shelter cat survey. Second, fecal samples were not obtained to determine whether *T. cati* or *Aelurostrongylus abstrusus* were present, both of which can cause pulmonary vascular lesions and radiographic signs. It is also possible that some of the cats had both heartworm and* T. cati* and *A. abstrusus* infections at some point. The third major limitation was the inability to fully explore the pulmonary vessels in the right caudal lung lobe and perform a complete necropsy to look for aberrant heartworms.

## Conclusions

Running multiple tests increases the likelihood of diagnosing an active FHWI and HARD but does not ensure it. While a positive HW Ab or HW Ag indicates a current or prior heartworm infection, a negative test result for both does not rule out FHWI or HARD. The ideal scenario would be a panel of tests for coughing cats that includes heartworm antibody tests that have been validated utilizing a model that more closely mimics the exposure cats receive in nature and a heat-treated HW Ag test. An argument could be made to include serology for *T. cati* and *A. abstrusus* as both parasites can lead to the same radiographic signs and pulmonary pathology. The addition of radiographs would help confirm findings suggested by serology.

While HARD is an elusive diagnosis given the reported shortcomings of the currently available tests, it may be obtainable in many cases by running a heat-treated HW Ag test and an existing HW Ab test. It should be noted neither the Heska nor Synbiotics test approaches the level of sensitivity reported utilizing experimentally infected cats. Based on the poor performance of the Synbiotics test in this study and the results of four other studies [6, 7, 23, 25], the Heska test would appear to be the preferred HW Ab test. Regardless of the results, all cats residing in areas endemic for heartworms should be placed on heartworm preventive therapy. The growing body of cumulative evidence indicates that cats are infected at the same rate as dogs and thus should be protected.

### Supplementary Information

**Additional file 1: Table S1.** Raw data from necropsy, serology, histopathology, and radiographs.

**Additional file 2: **Statistical calculations.

## Data Availability

All data generated or analyzed during this study are included in this published article.
