# Supplementary material for: Evaluation of feline heartworm disease based on gross necropsy, serology, pulmonary histopathology, and radiographic evidence in adult shelter cats in northeastern Alabama
Source: Parasit Vectors. 2024 Mar 29;17:161. doi: 10.1186/s13071-024-06178-9 (PMC10979576; doi:10.1186/s13071-024-06178-9)
Supplement: Supplementary file 1 — Additional file 1: Table S1. Raw data from necropsy, serology, histopathology, and radiographs. [file 13071_2024_6178_MOESM1_ESM.docx]

Table S1: Raw data from necropsy, serology, histopathology, and radiographs

| Case# | Necropsy | Synbiotics Ab Test kit | HWAb-1 | HWAb-2 | HWAg | Ag-Heat | Arteriole | Bronchiole | Radiographs | |
| --- | --- | --- | --- | --- | --- | --- | --- | --- | --- | --- |
| Q 09-13 |  | Negative | 0.4855* | 0.069 | Negative |  | 99.61% | 34.22% | 0 |  |
| Q 09-04 |  | Negative | 0.161 | 0.101 | Negative | Positive | 98.02% | 56.71% | 0.5 |  |
| Q 10-08 |  | Negative | 0.350* | 0.7155* | Positive | Positive | 97.59% | 48.22% | 2 |  |
| Q 09-10 |  | Negative | 0.099 | 0.137 | Negative |  | 97.40% | 93.24% | 3 |  |
| Q 10-13 |  | Negative | 0.064 | 0.081 | Negative |  | 97.14% | 59.02% | 1 |  |
| Q 10-12 | 1 F | Negative | 0.750* | 0.149 | Positive | Positive | 96.76% | 68.88% | 2 |  |
| Q 10-01 |  | Negative | 0.159 | 0.047 | Negative |  | 96.08% | 59.06% | 0.5 |  |
| Q 09-12 |  | Negative | 0.105 | 0.050 | Negative | Positive | 93.38% | 42.59% | 0.5 |  |
| Q 09-03 | 1 F & 1 M | Negative | 1.083* | 0.2105* | Positive | Positive | 92.37% | 44.70% | 1 |  |
| Q 09-15 |  | Negative | 0.169 | 0.061 | Negative |  | 92.05% | 45.82% | 0.5 |  |
| Q 09-16 |  | Negative | 0.134 | 0.098 | Negative |  | 89.35% | 55.68% | 1 |  |
| Q 10-11 |  | Negative | 0.062 | 0.050 | Negative |  | 78.43% | 47.40% | 1 |  |
| Q 09-02 |  | Negative | 0.498* | 0.041 | Negative | Positive | 77.11% | 42.39% | 1 |  |
| Q 10-24 |  | Negative | 0.070 | 0.083 | Negative | Positive | 75.50% | 24.07% | 2 |  |
| Q 09-18 |  | Negative | 0.158 | 0.055 | Negative |  | 69.85% | 34.71% | 0.5 |  |
| Q 10-05 |  | Negative | 0.103 | 0.057 | Negative |  | 69.57% | 44.71% | 0.5 |  |
| Q 09-01 |  | Negative | 0.195 | 0.2395* | Negative | Positive | 68.10% | 38.32% | 1 |  |
| Q 09-14 |  | Negative | 1.774* | 0.7735* | Negative |  | 67.54% | 34.39% | 0.5 |  |
| Q 10-06 |  | Negative | 0.067 | 0.045 | Negative |  | 67.31% | 51.56% | 0.5 |  |
| Q 10-26 |  | Negative | 0.060 | 0.045 | Negative |  | 64.78% | 27.45% | 0.5 |  |
| Q 09-19 |  | Negative | 0.055 | 0.044 | Negative |  | 64.18% | 25.36% | 0.5 |  |
| Q 09-07 |  | Negative | 0.237* | 0.055 | Negative |  | 62.67% | 48.33% | 1 |  |
| Q 10-09 | 2 Fragments | Negative | 0.136 | 0.111 | Negative |  | 62.50% | 38.56% | 0.5 |  |
| Q 10-07 |  | Negative | 0.8215* | 0.3275* | Negative |  | 61.96% | 53.19% | 0 |  |
| Q 09-06 |  | Negative | 0.227* | 0.067 | Negative |  | 59.81% | 47.91% | 0 |  |
| Q 10-16 |  | Negative | 0.5485* | 0.438* | Negative |  | 56.98% | 48.63% | 0 |  |
| Q 10-28 |  | Negative | 3.482* | 0.5225* | Negative | Positive | 54.45% | 40.14% | 2 |  |
| Q 10-23 |  | Negative | 1.161* | 1.0745* | Negative |  | 50.59% | 39.61% | 0.5 |  |
| Q 10-22 |  | Negative | 0.115 | 0.046 | Negative |  | 43.36% | 39.04% | 0 |  |
| Q 09-17 |  | Negative | 0.094 | 0.078 | Negative |  | 41.79% | 29.57% | 0 |  |
| Q 10-04 |  | Negative | 0.048 | 0.054 | Negative |  | 39.87% | 44.44% | 0.5 |  |
| Q 10-30 |  | Negative | 0.068 | 0.288* | Negative |  | 38.59% | 33.47% | 1 |  |
| Q 10-21 |  | Negative | 0.059 | 0.079 | Negative |  | 38.21% | 29.78% | 0.5 |  |
| Q 09-09 |  | Negative | 0.054 | 0.055 | Negative |  | 36.62% | 62.53% | 0.5 |  |
| Q 10-10 |  | Negative | 0.2185* | 0.861* | Negative |  | 35.59% | 28.82% | 0.5 |  |
| Q 10-03 |  | Negative | 0.108 | 0.3995* | Negative |  | 35.41% | 38.26% | 0.5 |  |
| Q 09-11 |  | Negative | 0.065 | 0.070 | Negative |  | 35.02% | 30.69% | 0 |  |
| Q 10-29 |  | Negative | 0.114 | 0.094 | Negative |  | 33.97% | 22.48% | 0.5 |  |
| Q 09-20 |  | Negative | 0.054 | 0.049 | Negative |  | 33.41% | 34.15% | 0 |  |
| Q 09-05 |  | Negative | 0.055 | 0.073 | Negative |  | 32.51% | 36.89% | 0 |  |
| Q 09-21 |  | Negative | 0.046 | 0.060 | Negative |  | 31.89% | 31.69% | 0.5 |  |
| Q 10-25 |  | Negative | 0.052 | 0.054 | Negative |  | 31.89% | 26.84% | 0.5 |  |
| Q 10-15 |  | Negative | 0.056 | 0.055 | Negative |  | 31.39% | 44.52% | 0 |  |
| Q 10-27 |  | Negative | 0.048 | 0.075 | Negative |  | 31.33% | 20.65% | 0 |  |
| Q 10-14 |  | Negative | 0.053 | 0.059 | Negative | Positive | 29.59% | 30.22% | 0.5 |  |
| Q 10-20 |  | Negative | 0.058 | 0.053 | Negative |  | 27.02% | 21.72% | 0.5 |  |
| Q 09-08 |  | Negative | 0.071 | 0.077 | Negative |  | 26.31% | 42.33% | 0 |  |
| Q 10-18 |  | Negative | 0.048 | 0.151 | Negative |  | 24.82% | 27.71% | 0.5 |  |
| Q 10-02 |  | Negative | 0.088 | 0.091 | Negative |  | 23.59% | 38.86% | 0.5 |  |
| Q 10-17 |  | Negative | 0.068 | 0.069 | Negative |  | 15.05% | 12.60% | 0.5 |  |

Data sorted by Arteriole wall to lumen ratio.

*Indicates a positive antibody response.
