# Supplementary material for: Evaluation of feline heartworm disease based on gross necropsy, serology, pulmonary histopathology, and radiographic evidence in adult shelter cats in northeastern Alabama
Source: Parasit Vectors. 2024 Mar 29;17:161. doi: 10.1186/s13071-024-06178-9 (PMC10979576; doi:10.1186/s13071-024-06178-9)
Supplement: Supplementary file 2 — Additional file 2: Statistical calculations. [file 13071_2024_6178_MOESM2_ESM.docx]

| Fit Statistics | |
| --- | --- |
| -2 Res Log Likelihood | 379.05 |
| AIC (smaller is better) | 381.05 |
| AICC (smaller is better) | 381.14 |
| BIC (smaller is better) | 382.96 |
| CAIC (smaller is better) | 383.96 |
| HQIC (smaller is better) | 381.77 |
| Generalized Chi-Square | 46.00 |
| Gener. Chi-Square / DF | 1.00 |

| Type III Tests of Fixed Effects | | | | | | |
| --- | --- | --- | --- | --- | --- | --- |
| Effect | Num DF | Den DF | Chi-Square | F Value | Pr > ChiSq | Pr > F |
| HW_Ab1 | 1 | 46 | 32.62 | 32.62 | <.0001 | <.0001 |
| HW_Ab2 | 1 | 46 | 7.34 | 7.34 | 0.0067 | 0.0094 |
| HW_Ag  (heat treated) | 1 | 46 | 4.02 | 4.02 | 0.0449 | 0.0508 |

Model without Radiographs

| R-Square Type Goodness-of-Fit Information |
| --- |

| MODEL FITTING INFORMATION |
| --- |

| DESCRIPTION | Value |
| --- | --- |
| Total Observations | 50 |
| N (number of subjects) | 50 |
| Number of Fixed-Effects Parameters | 4 |
| Average Model R-Square: | 0.419454 |
| Average Model Adjusted R-Square: | 0.368972 |
| Average Model Concordance Correlation: | 0.591008 |
| Average Model Adjusted Concordance Correlation: | 0.555443 |
| Variance-Covariance Concordance Correlation: | 0.964858 |
| Discrepancy Function | 0.16933 |
| s = Rank of robust sandwich estimator, OmegaR | 4 |
| s1 = Number of unique non-zero off-diagonal elements of OmegaR | 6 |
| Approx. Chi-Square for H0: Covariance Structure is Correct | 8.466484 |
| DF1 = s(s+1)/2 | 10 |
| Pr > Chi Square based on degrees of freedom, DF1 | 0.583369 |
| DF2 = s+s1, modified degrees of freedom | 10 |
| Pr > Chi Square based on modified degrees of freedom, DF2 | 0.583369 |

| Fit Statistics | |
| --- | --- |
| -2 Res Log Likelihood | 371.66 |
| AIC (smaller is better) | 373.66 |
| AICC (smaller is better) | 373.76 |
| BIC (smaller is better) | 375.58 |
| CAIC (smaller is better) | 376.58 |
| HQIC (smaller is better) | 374.39 |
| Generalized Chi-Square | 45.00 |
| Gener. Chi-Square / DF | 1.00 |

| Type III Tests of Fixed Effects | | | | | | |
| --- | --- | --- | --- | --- | --- | --- |
| Effect | Num DF | Den DF | Chi-Square | F Value | Pr > ChiSq | Pr > F |
| HW_Ab1 | 1 | 45 | 31.75 | 31.75 | <.0001 | <.0001 |
| HW_Ab2 | 1 | 45 | 10.76 | 10.76 | 0.0010 | 0.0020 |
| HW_Ag  (heat treated) | 1 | 45 | 0.09 | 0.09 | 0.7695 | 0.7708 |
| Radiographs | 1 | 45 | 8.95 | 8.95 | 0.0028 | 0.0045 |

Model including Radiographs.

| R-Square Type Goodness-of-Fit Information |
| --- |

| MODEL FITTING INFORMATION |
| --- |

| DESCRIPTION | Value |
| --- | --- |
| Total Observations | 50 |
| N (number of subjects) | 50 |
| Number of Fixed-Effects Parameters | 5 |
| Average Model R-Square: | 0.529261 |
| Average Model Adjusted R-Square: | 0.476957 |
| Average Model Concordance Correlation: | 0.692179 |
| Average Model Adjusted Concordance Correlation: | 0.657976 |
| Variance-Covariance Concordance Correlation: | 0.954747 |
| Discrepancy Function | 0.351073 |
| s = Rank of robust sandwich estimator, OmegaR | 5 |
| s1 = Number of unique non-zero off-diagonal elements of OmegaR | 10 |
| Approx. Chi-Square for H0: Covariance Structure is Correct | 17.55367 |
| DF1 = s(s+1)/2 | 15 |
| Pr > Chi Square based on degrees of freedom, DF1 | 0.286857 |
| DF2 = s+s1, modified degrees of freedom | 15 |
| Pr > Chi Square based on modified degrees of freedom, DF2 | 0.286857 |
